# Factors associated with in-office influenza vaccination by U.S. pediatric providers

**DOI:** 10.1186/1471-2431-13-180

**Published:** 2013-11-06

**Authors:** Chyongchiou Jeng Lin, Mary Patricia Nowalk, Seth L Toback, Christopher S Ambrose

**Affiliations:** 1 1802 Windham Court, Sewickley, PA 15143, USA; 2 4715 Baldwin Manor Road, Pittsburgh, PA 15227, USA; 3 Former employee of MedImmune, One MedImmune Way, Gaithersburg, MD 20878, USA; 4 Medical and Scientific Affairs, Infectious Disease, MedImmune, One MedImmune Way, Gaithersburg, MD 20878, USA

**Keywords:** Pediatrician, Seasonal influenza vaccine, Coverage, Compliance

## Abstract

**Background:**

In the United States, influenza vaccination is recommended for all children 6 months and older; however, vaccination rates are below target levels. A broad sample of U.S. pediatric offices was assessed to determine factors that influence in-office influenza vaccination rates.

**Methods:**

Offices (N = 174) were recruited to participate in an observational study over three influenza seasons (2008–2009, 2009–2010, 2010–2011). Only data from the first year of an office’s participation in the study were used. Associations of coverage and 2-dose compliance rates with office characteristics and selected vaccination activities were examined using univariate regression analyses and linear regression analyses using office characteristics identified a priori and vaccination activities with *P* values ≤0.10 in univariate analyses.

**Results:**

Influenza vaccination coverage for children 6 months to 18 years of age averaged 25.2% (range: 2.0%–69.1%) and 2-dose compliance for children <9 years of age averaged 53.4% (range: 5.4%–96.2%). Factors associated with increased coverage were non-rural site (*P* = 0.025), smaller office size (fewer than 5000 patients; *P* < 0.001), use of evening and weekend hours to offer influenza vaccine (*P* = 0.004), a longer vaccination period (*P* = 0.014), and a greater influenza vaccine coverage rate among office staff (*P* = 0.012). Increased 2-dose compliance was associated with smaller office size (*P* = 0.001) and using patient reminders (*P* = 0.012) and negatively related to use of electronic provider reminders to vaccinate (*P* = 0.003).

**Conclusions:**

To maximize influenza vaccine coverage and compliance, offices could offer the vaccine during evening and weekend hours, extend the duration of vaccine availability, encourage staff vaccination, and remind patients that influenza vaccination is due. Additional efforts may be required in large offices and those in rural locations.

## Background

In the last decade, the U.S. Advisory Committee on Immunization Practices (ACIP) has incrementally widened the annual influenza vaccination recommendations such that currently, influenza vaccination is recommended for all individuals ≥6 months [1]. As a result, uptake of influenza vaccine among children has increased significantly; however, U.S. vaccination rates are below target levels of 80% [1] and disparities in coverage exist across socioeconomic [2] and racial groups [3].

One hindrance to reaching the target influenza vaccination rates in the U.S. may be the breadth of office changes required to vaccinate all eligible children and the magnitude of implementing those changes in offices across the country. The fact that influenza vaccine is administered annually and seasonally, rather than on an age-based schedule, requires offices to anticipate the demand for vaccine up to six months in advance and plan for administration over a defined time period. Previous research has suggested that an additional 42 to 49 million office visits would be required to vaccinate children 5 to 18 years of age against influenza; the burden was lower with use of a longer vaccination period [4]. Further complicating the situation is the fact that first-time vaccine recipients younger than 9 years should receive two doses of influenza vaccine at least one month apart [1].

Efficient and effective methods for vaccinating large numbers of children in primary care offices are essential. Previous research in this arena has been limited geographically to one or a few offices, to offices in a localized area [5-10], or to practices in three diverse U.S. counties [11]. The purpose of this study was to describe influenza vaccination activities in 174 pediatric offices across the U.S. in a variety of settings and examine the relationships of office characteristics and those activities to influenza vaccination coverage and two-dose compliance.

## Methods

Data were collected through a prospective, observational study conducted at outpatient pediatricians’ offices in the U.S. during the 2008–2009, 2009–2010, and 2010–2011 influenza seasons. A central institutional review board (Coast IRB, LLC, Lake Forest, CA; protocol MI-MA156) approved that the study could be exempted clinical research in accordance with Health Insurance Portability and Accountability Act of 1996 (HIPAA; Public Law 104–191) privacy rules and the Code of Federal Regulations (45 CFR §46.101). Study methods have been described previously [12,13] and are summarized below.

Inclusion criteria for primary care practices treating children were provision of influenza vaccine at the office’s location and the ability to generate an accurate count of their office patient population by age. Each season, a random sample of pediatricians from the American Medical Association list of physicians was invited to participate; offices from prior seasons were eligible to continue in the study. Practices were selected to achieve a geographically balanced sample and received limited financial compensation for study-related data collection and submission. Because participation in the study for multiple years may have changed influenza vaccination activities, data from the first season of participation for 174 offices were used for these analyses; 65 collected data in 2008–2009, 43 in 2009–2010, and 66 in 2010–2011.

Survey information including total number of patients, office demographics, influenza vaccine supplies, and dates of administration was completed by a single contact in the office at the beginning and end of the season. Twice monthly, from August 1 through March 31, offices recorded in a web-based electronic database: 1) each influenza vaccination administered by age group (6–23 months, 24–59 months, 5–8 years, and 9–18 years), first or second vaccination for the child, type of vaccine (multi-dose injectable, prefilled injectable, or intranasal spray), and by payor (commercial insurance, Medicaid with Vaccines for Children (VFC), Medicaid without VFC, self-pay); 2) office efforts to increase influenza vaccine uptake, including verbal recommendations to patients, in-office reading materials, posters, videotapes, letters, phone calls, on-hold recordings, emails, and office-provided patient incentives (e.g., toys, stickers, and coupons); 3) staff activities (comparing patient vaccination rates among staff, computerized vaccination reminders to staff, and educational workshops related to influenza vaccination); and 4) community events (school vaccination programs, mobile vaccine clinics, clinics at other locations, and local media coverage).

### Statistical analysis

Survey data were summarized with descriptive statistics for all offices. Coverage (proportion of children receiving ≥1 dose of influenza vaccine) and 2-dose compliance (of the children identified as needing 2 doses, the proportion who received 2 doses) were calculated. During the 2009–2010 pandemic season, only data from seasonal influenza vaccines were assessed. Similar vaccination activities were combined; for example, patient reminders by voicemail, email, and mailed notices = “reminders”, and total hours during which vaccine was offered outside of normal office hours = “evening/weekend hours”. Univariate analyses were performed to determine which vaccination promotion activities were associated with influenza vaccination coverage and compliance. Linear regression analyses for each outcome variable were performed using the office characteristics identified a priori and those vaccination activities with *P* values ≤0.10 in univariate analyses. All independent variables were entered simultaneously. Influenza season was not significantly related to either coverage or compliance while accounting for the other variables. Hence, data for all three years were combined for subsequent analyses. Statistical significance was set at alpha <0.05.

## Results

Characteristics of offices participating in the study such as location, size, staff to provider ratios, age distribution of children, and proportion of low income children (as indicated by participation in the VFC program) varied widely across offices, consistent with a broad representation of offices across the country (Table 1). Likewise, there was variability in efforts to promote influenza vaccination and in vaccination rates. The average coverage for children 6 months to 18 years of age was estimated at 25.2%. Compared with patients aged 9 years and older, those younger than 9 years had a higher coverage rate (32.2% vs. 18.6%). The 2-dose compliance for children younger than 9 years was estimated at 53.4%.

**Table 1:** Characteristics and activities to promote influenza vaccination of participating pediatric offices

**Characteristics and activities**	**Overall (N = 174)**
*Characteristics*	
Total staff, mean (range)	12.6 (2–60)
Ratio of staff (Nurse + Other) to Provider (MD + NP/PA), mean (range)	2.35 (0.14–9.00)
Total patients, mean (range)	6693 (525–36,531)
Total patient size ≥5000, n (% of offices)	85 (48.9)
Percentage of patients by age group	
6–23 months, % (range)	12.5 (2.0–42.8)
24–59 months, % (range)	21.1 (5.7–41.0)
5–8 years, % (range)	22.7 (2.9–54.2)
9–18 years, % (range)	43.7 (7.5–81.2)
Staff per 1000 Patients, mean (range)	2.7 (0.4–15.2)
Location, n (% of offices)	
Urban	33 (19.0)
Suburban	116 (66.7)
Rural	25 (14.4)
Percentage of Medicaid patients in office (SD)	28.3 (25.8)
Percentage of staff vaccinated (SD)	86.0 (21.7)
*Activities*	
Number of patient reminders used: (mail/phone, email, videotape, “on-hold” messages, mean (SD)	
0	72 (41.4)
1	56 (32.2)
2	35 (20.1)
3	9 (5.2)
4	2 (1.1)
Offer incentives (% of offices)	28.7
Family members offered vaccines (% of offices)	47.1
Number of total weekend/evening hours for influenza vaccination, mean (SD)	7.6 (21.5)
Offered weekend/evening hours for influenza vaccination (% of offices)	32.8
Comparison of vaccination rates within office (% of offices)	35.6
Electronic reminders to provider to vaccinate (% of offices)	28.2
Local immunization activities and media coverage of influenza vaccination occurred in community (% of offices)	54.6
Standing order program for influenza vaccine (% of offices)	59.8
Clinical staff education workshops during influenza season (% of offices)	22.4
Duration vaccine available, mean days (SD)	205.6 (60.8)
Coverage: % of children receiving ≥1 dose, mean (SD)	25.2 (14.9)
Coverage: % of children ≥9 years of age receiving ≥1 dose, mean (SD)	18.6 (14.8)
Coverage: % of children <9 years of age receiving ≥1 dose, mean (SD)	32.2 (18.8)
Compliance: % of first-time vaccinees <years of age receiving two doses, mean (SD)	53.4 (20.0)

MD = medical doctor; NP = nurse practitioner; PA = physician assistant.

In univariate analyses (data not shown), increasing access to influenza vaccine by extending the time vaccine was available and offering influenza vaccination opportunities during evening and weekend hours were related to increased vaccine coverage. Similarly, those two patient access variables, as well as patient and provider reminders, were significantly related to 2-dose compliance rates. In the final linear regression analysis (Table 2), vaccination coverage increased with increased ratio of office staff per 1000 patients (*P* < 0.001), greater uptake of vaccine by office staff (*P* = 0.020), duration of vaccine availability (0.6% increase for every additional 10 days that vaccine was offered; *P* = 0.014), and offering evening/weekend hours for vaccination (*P* = 0.004). Larger offices (>5000 patients) and those located in rural areas had lower influenza vaccine coverage (*P* < 0.05). Compliance was lower among larger offices (*P* = 0.001), those with more Medicaid-insured children, and those using electronic provider reminders to vaccinate (*P* < 0.05); compliance was higher with use of patient reminders (*P* = 0.012).

**Table 2:** Variables related to coverage and compliance with influenza vaccine in pediatric offices by linear regression

**Explanatory variables**	**Coverage**			**2-dose compliance**		
	**Coefficient**	**Standard error**	***P *** **value**	**Coefficient**	**Standard error**	***P *** **value**
(Constant)	1.89	6.76	0.781	54.92	7.34	<0.001
Rural vs suburban and urban	−6.21	2.75	**0.025**	−4.75	4.52	0.295
Office size ≥5000 patients	−11.20	2.02	**<0.001**	−11.33	3.31	**0.001**
Staff/1000 patients	2.06	0.44	**<0.001**	−1.06	0.72	0.146
% Medicaid patients	0.03	0.04	0.450	−0.131	0.062	**0.034**
% Staff vaccinated	0.10	0.04	**0.020**	0.094	0.070	0.181
Offered weekend/evening hours for vaccination	5.92	2.03	**0.004**	3.87	3.45	0.264
Duration vaccine available	0.06	0.02	**0.014**	—	—	—
Patient reminders used	—	—	—	8.66	3.39	**0.012**
Computer reminder to staff to vaccinate	—	—	—	−10.80	3.64	**0.003**

Values in boldface are statistically significant.

## Discussion

The majority of childhood vaccines are given according to an age-based schedule. This predictability allows primary care offices to develop routine office systems for providing vaccines and scheduling well child visits. Conversely, providing seasonal influenza vaccines requires an annual effort to prepare office operational systems, remind patients and providers, order additional supplies, and anticipate staffing needs. An office’s efforts may range from minimal to significant office system restructuring in order to accommodate its patient population, as well as adjust to seasonal conditions such as vaccine supply and temporal availability. The need to vaccinate first-time vaccinees twice, separated by at least a 4-week interval, further complicates offices’ operations during this part of the year.

A previous study of pediatric offices in three counties across the U.S. [11] reported that suburban location, lower patient volume, and offering evening and/or weekend express vaccination services were associated with higher influenza vaccination rates among children 6 to 23 months of age. In the present study of children 6 months to 18 years of age, similar associations were found, with higher coverage rates among suburban/urban offices compared with rural offices, those with fewer total patients, larger staff to patient ratios, more staff who were vaccinated against influenza and offices that offered more evening/weekend hours for children to receive influenza vaccine. Having an office visit between October and January was associated with higher vaccination rates [11]; we found that a longer duration of vaccine availability was significantly related to higher vaccination rates. For offices with large patient populations, it seems more sensible to extend the vaccination season beyond January to more easily accommodate large numbers of children. Influenza vaccination is being extended into earlier, but not later months of the influenza season [13], but physicians have reported that the need to administer the second dose of vaccine for young, first-time vaccinees is an impediment to providing late season vaccinations [14].

A recent meta-analysis of interventions to increase influenza vaccination rates among community-dwelling adults found that financial incentives for patients, strategies for providers including audit and feedback, financial incentives, and reminders, and system strategies such as standing orders were most effective [15]. A qualitative analysis of barriers and facilitators of influenza vaccination for children suggested provider recommendation and convenient access in which parents would not have to miss work to have a child vaccinated, standing order protocols, vaccination clinics, and ensuring staff support of the vaccination effort [16]. Combined with these previous studies, our results suggest that increasing the number of staff, especially in larger offices to: 1) handle greater demand during influenza season; 2) cover evening and weekend hours and; 3) extend the vaccination period would enable offices to improve influenza vaccination coverage. Furthermore, staff support of influenza vaccination demonstrated by receiving the vaccine themselves can be an important form of provider recommendation.

Little research has been performed on factors associated with 2-dose influenza vaccination compliance. In an earlier analysis of the 2010–2011 results from the current study, Toback et al. [17] found that 2-dose compliance was higher in smaller offices and in offices with video reminder messages in waiting rooms. Other research among 6–23-month-old children found only visits between October and January related to increased levels of compliance [11]. In the current, multi-year analysis, higher 2-dose compliance rates were related to smaller office size, use of patient reminders, and caring for fewer Medicaid insured children. The types and frequency of reminders needed to reach low income children may differ from those for more affluent, privately insured children. Recent studies have reported that patient reminders that resulted in more frequent attendance at health care settings were associated with higher influenza vaccination [10], and that text messaging was found to be an effective method for reminding low income urban children about influenza vaccination [8]. However, the latter study did not specifically target or report 2-dose compliance, and overall influenza vaccination coverage remained low.

The use of computerized provider reminders to vaccinate children against influenza was associated with lower compliance rates. We have no explanation for this finding except for anecdotal reports that providers can become reminder-fatigued and may turn off or just ignore these prompts, or perhaps, the electronic prompts have not been programmed appropriately for vaccinees requiring a second dose. Further research in this area is clearly needed.

### Strengths and limitations

One of the strengths of this study is the large number of participating pediatric offices from across the U.S., presumably representing the broad spectrum of types and location of offices as well as the assessment of a diverse array of strategies to enhance influenza vaccination. Although data from this study were previously analyzed in a qualitative manner, the current analysis represents the largest and most robust statistical analysis of office-level factors associated with pediatric influenza vaccination. Further, 2-dose compliance to influenza vaccination is seldom addressed in the literature and this study examines factors related to this important aspect of influenza vaccine uptake in children. The primary limitation is that the offices enrolled represent a convenience sample; their representativeness of U.S. pediatric offices overall is unknown. Additionally, vaccination denominators and numerators were self-reported and included only vaccinations given in the office; vaccinations outside of the office were not documented. Thus, the effect of efforts to educate and inform parents/patients may have been underreflected in the number of in-office vaccinations given.

## Conclusions

To maximize vaccine coverage, pediatricians’ offices should offer vaccine during evening and weekend hours and extend the duration of vaccine availability. Offices may also be able to achieve higher influenza vaccination coverage with a higher staff to patient ratio. Patient reminder systems should be employed to help maximize 2-dose compliance. Additional efforts may be required in large offices and those in rural locations.

## Consent

The observational data in this study were not collected from patients. Hence, no consent from patients was necessary.

## Abbreviations

ACIP: Advisory Committee on Immunization Practices; NVSN: New Vaccine Surveillance Network; VFC: Vaccines for Children.

## Competing interests

CJL has served as a consultant for MedImmune. MPN has served as a consultant for MedImmune, an influenza vaccine manufacturer and has received grant funding from MedImmune and Merck & Co, Inc., vaccine manufacturers. SLT was an employee of MedImmune at the time of the study. CSA is an employee of MedImmune and holds stock or stock options in AstraZeneca, the parent company of MedImmune. The authors declare no other competing interests.

## Authors’ contributions

CSA conceptualized and designed the study; analyzed and interpreted the data; critically revised the manuscript for important intellectual content; and approved the final manuscript as submitted. SLT conceptualized and designed the study; analyzed and interpreted the data; critically revised the manuscript for important intellectual content; and approved the final manuscript as submitted. CJL acquired, analyzed, and interpreted the data; critically revised the manuscript for important intellectual content; and approved the final manuscript as submitted. MPN conceptualized and designed the analyses; acquired, analyzed, and interpreted the data; drafted and critically revised the manuscript for important intellectual content; and approved the final manuscript as submitted.

## Pre-publication history

The pre-publication history for this paper can be accessed here:

http://www.biomedcentral.com/1471-2431/13/180/prepub

## References

[B1] Centers for Disease Control and Prevention Prevention and control of influenza with vaccines: recommendations of the Advisory Committee on Immunization Practices (ACIP), 2010 MMWR 2010 59 1 62 20689501

[B2] Yoo BK Berry A Kasajima M Szilagyi PG Association between Medicaid reimbursement and child influenza vaccination rates Pediatrics 2010 126 e998 e1010 10.1542/peds.2009-3514 20956412

[B3] Centers for Disease Control and Prevention Prevention and control of influenza with vaccines: recommendations of the Advisory Committee on Immunization Practices (ACIP)--United States, 2012–13 influenza season MMWR Morb Mortal Wkly Rep 2012 61 613 618 22895385

[B4] Rand CM Szilagyi PG Yoo BK Auinger P Albertin C Coleman MS Additional visit burden for universal influenza vaccination of US school-aged children and adolescents Arch Pediatr Adolesc Med 2008 162 1048 1055 10.1001/archpedi.162.11.1048 18981353

[B5] Dombkowski KJ Harrington LB Dong S Clark SJ Seasonal influenza vaccination reminders for children with high-risk conditions: a registry-based randomized trial Am J Prev Med 2012 42 71 75 10.1016/j.amepre.2011.09.028 22176850

[B6] O’Connor ME Everhart RM Berg M Federico SG Hambidge SJ Pediatric influenza immunization in an integrated safety net health care system Vaccine 2012 30 2951 2955 10.1016/j.vaccine.2012.02.060 22401868

[B7] Paul IM Eleoff SB Shaffer ML Bucher RM Moyer KM Gusic ME Improving influenza vaccination rates for children through year-round scheduling Ambul Pediatr 2006 6 230 234 10.1016/j.ambp.2006.04.006 16843256

[B8] Stockwell MS Kharbanda EO Martinez RA Vargas CY Vawdrey DK Camargo S Effect of a text messaging intervention on influenza vaccination in an urban, low-income pediatric and adolescent population: a randomized controlled trial JAMA 2012 307 1702 1708 10.1001/jama.2012.502 22535855

[B9] Szilagyi PG Albertin C Humiston SG Rand CM Schaffer S Brill H Stankaitis J Yoo BK Blumkin A Stokley S A randomized trial of the effect of centralized reminder/recall on immunizations and preventive care visits for adolescents Acad Pediatr 2013 13 204 213 10.1016/j.acap.2013.01.002 23510607 PMC4594853

[B10] Uwemedimo OT Findley SE Andres R Irigoyen M Stockwell MS Determinants of influenza vaccination among young children in an inner-city community J Community Health 2012 37 663 672 10.1007/s10900-011-9497-9 22045471

[B11] Poehling KA Fairbrother G Zhu Y Donauer S Ambrose S Edwards KM Staat MA Prill MM Finelli L Allred NJ Bardenheier B Szilagyi PG New Vaccine Surveillance Network Practice and child characteristics associated with influenza vaccine uptake in young children Pediatrics 2010 126 665 673 10.1542/peds.2009-2620 20819893 PMC3673003

[B12] Bhatt P Block SL Toback SL Ambrose CS A prospective observational study of US in-office pediatric influenza vaccination during the 2007 to 2009 influenza seasons: use and factors associated with increased vaccination rates Clin Pediatr (Phila) 2010 49 954 963 10.1177/0009922810370868 20522609

[B13] Toback SL Herley J Edelman L Ambrose CS Trends in U.S. pediatric influenza vaccination from 2006 to 2010 among children with private insurance Vaccine 2011 29 4225 4229 10.1016/j.vaccine.2011.03.108 21497635

[B14] Suh C McQuillan L Daley MF Crane LA Beaty B Barrow J Babbel C Dickinson LM Kempe A Late-season influenza vaccination: a national survey of physician practice and barriers Am J Prev Med 2010 39 69 73 10.1016/j.amepre.2010.03.010 20547279

[B15] Lau D Hu J Majumdar SR Storie DA Rees SE Johnson JA Interventions to improve influenza and pneumococcal vaccination rates among community-dwelling adults: a systematic review and meta-analysis Ann Fam Med 2012 10 538 546 10.1370/afm.1405 23149531 PMC3495928

[B16] Bhat-Schelbert K Lin CJ Matambanadzo A Hannibal K Nowalk MP Zimmerman RK Barriers to and facilitators of child influenza vaccine - perspectives from parents, teens, marketing and healthcare professionals Vaccine 2012 30 2448 2452 10.1016/j.vaccine.2012.01.049 22300721

[B17] Toback SL Rothstein E Bhatt P Carr W Ambrose CS In-office influenza vaccination by US pediatric providers varies greatly and is higher among smaller offices Clin Pediatr 2012 51 551 559 10.1177/0009922812443731 22589476

